# Impact of Confinement on Coping Strategies and Psychosocial Factors among University Students

**DOI:** 10.3390/ejihpe12080067

**Published:** 2022-08-01

**Authors:** Carla Gutiérrez-Lozano, Inmaculada García-Martínez, José María Augusto-Landa, Samuel P. León

**Affiliations:** 1 Department of Didactics and School Organization, Faculty of Education, Campus de Cartuja, University of Granada, s/n, 18071 Granada, Spain; cargutloz@correo.ugr.es; 2 Department of Psychology, Campus las Lagunillas, University of Jaén, s/n, 23071 Jaén, Spain; jaugusto@ujaen.es; 3 Department of Pedagogy, Campus las Lagunillas, University of Jaén, s/n, 23071 Jaén, Spain; sparra@ujaen.es

**Keywords:** coping strategies, emotional intelligence, anxiety, university students, COVID-19

## Abstract

The pandemic has had psychological effects on the university population. Factors such as emotional intelligence, coping strategies and levels of anxiety, depression and stress have been affected by the situation generated by COVID-19. This study aims to analyze how EI, coping strategies and levels of anxiety, depression and stress have been affected by the situation generated by the pandemic in a population of 567 students from the University of Jaén (Spain). For this purpose, we administered three instruments: the Wong and Law emotional intelligence scale (WLEIS), the Spanish version of the coping strategies inventory (CSI) and the depression anxiety stress scales (DASS-21). At the same time, we asked students to describe their personal circumstances during confinement and their tendency to follow the measures and recommendations promoted by the Ministry of Health. The results obtained showed a positive relationship between EI and coping strategies and a negative relationship with levels of depression, anxiety and stress. A positive relationship was also found between coping and levels of anxiety, depression and stress. It was also found that the circumstances in which students experienced the period of confinement also modulated their levels of EI, coping strategies and their levels of depression, anxiety and stress.

## 1. Introduction

In March 2020, Spain was fully involved in a worldwide pandemic caused by an infection caused by COVID-19, which originated in the city of Wuhan (China) in December 2019 [1]. According to WHO [2], the virus that causes the COVID-19 illness has affected 213 countries and territories worldwide with 14 million cases and half a million deaths. In Spain, according to the Secretary of State for Health (Update No. 336), as of 22 March 2021, a total of 3,228,803 people has been confirmed infected and 73,543 have died since the beginning of the pandemic. On 11 March 2020, the Spanish Government decreed a State of Health Alert in order to protect the population from this infection in order to halt the spread of the COVID-19 virus [3]. Among the different measures that were implemented in Spain, mandatory social isolation of the population (which lasted 3 months), produced significant psychological consequences [4], the most frequent of which include stress, anxiety, loneliness, depression and emotional exhaustion [5,6]. These psychological consequences are still ongoing, and they are maintained in part by the prevalence of uncertainty, health risks of infection and the over-information to which people are exposed [7]. In a systematic review by Xiong et al. [8] the authors found high incidence rates of anxiety symptoms, depression, post-traumatic stress disorder, psychological distress and stress in the general population during the COVID-19 period in different countries, such as China, Spain, Italy, Iran, USA, Turkey, Nepal and Denmark. The timing of the COVID-19 pandemic may have been emotionally challenging and stressful for all affected individuals, and particularly for those subgroups of the population at higher risk of mental health problems. One of these vulnerable groups is university students [9]. Although the real impact of the COVID-19 on the educational achievement and mental health of university students is still unknown, it is expected to be highly meaningful [10,11]. 

Within this context, the construct of emotional intelligence (EI, hereafter), understood as an interrelated range of skills to identify, use, understand and manage the own emotions and those of others [12], takes on particular relevance. As stated by some authors, EI provides a theoretical framework for addressing the role of emotional skills in the process of coping and interpersonal functioning in times of crisis [13]. 

### 1.1. Emotional Intelligence and Coping Strategies

The concept of EI and its definition have been widely discussed and argued from both the field of education and psychology, with different approaches [14,15,16]. In the scientific literature, two opposing models have been identified: the ability model, which considers EI to be a genuine intelligence that allows the person to adapt to the context, and the mixed models, where EI is understood as a set of traits that can vary more or less over time and that determines the person’s behavior. This paper adheres to the concept of mixed models; specifically, the proposal developed by Mayer and Salovey [17], who argue that EI lies between the emotional and the cognitive, and may be defined as the ability to perceive, assimilate, understand and manage one’s own emotions, as well as the ability to detect and interpret the emotions of others.

On the other hand, with regard to the concept of coping, an accepted definition is provided by Folkman and Lazarus [18], which defines it as those efforts, both cognitive and behavioral, made by a person to manage, reduce or accept the internal or external demands generated by a stressful situation, suggesting that these responses generated in relation to the environment are influenced by cognitive appraisal processes, thus modulating the adaptive consequences in each of the situations.

Coping can result in different types of responses, which leads to a distinction between different classifications, behaviors aimed at modifying the situation causing the stress and those aimed at the emotion produced by the stressful situation [19]. According to the type of processes involved, we find, among others, cognitive coping, which consists of addressing the situation with behaviors such as cognitive restructuring, cognitive reinterpretation of the situation, directing attention to other issues or carrying out social comparison processes. Regardless of the type of response, coping strategies can generally be considered adaptive (reducing stress and promoting health in the long term) or non-adaptive (reducing stress in the short term but impairing health in the long term) [20]. As Prentice et al. [21] point out, in the case of COVID-19, the virus itself acts as a health stressor and causes subjects to take both medical and non-medical measures in an attempt to reduce the likelihood of infection. In addition to these measures taken by the subjects, the restrictive measures imposed by governments, in the case of Spain, by the Autonomous Communities, produce different types of coping, depending on the person, such as emotion-oriented (focusing on the uncertainty of its duration) or task-oriented (seeing this period as an opportunity to share time with the people living with them). As some authors [22,23] point out, the type of strategies depends to a large extent on the personality traits and the EI of the subjects.

### 1.2. Psychosocial Variables and Confinement by COVID-19

The situation derived from the pandemic and the confinement as a measure to control the spread of the virus generated the appearance of different factors considered stressors, such as: the changes produced in people’s daily routines, the humanitarian and economic crisis, the difficulties that have developed in companies, etc., together with the distancing, social isolation and the fear of contagion or death due to the disease [24]. All these factors show that, in addition to attacking physical health, the appearance of the COVID-19 virus and the resulting situations also had an impact on the psychological state and emotional wellbeing of people, who were affected by negative feelings such as fear, uncertainty, frustration, anxiety, sadness and worry [25]. This is a problem, as it has been shown in situations that developed in the wake of other pandemics that these effects do not necessarily disappear after the current situation, but may persist over time [26]. For all these reasons, it is essential to carry out studies that provide reliable data on the subject in order to be able to study in depth the psychological effects, as well as possible measures to be taken to promote good individual and community mental health after these abrupt changes in people’s daily lives [27].

So far, studies carried out in different countries on the psychological impact of the pandemic on various population groups show results that confirm this. This is the case of the study conducted by Wang et al. [28] with 1210 Chinese citizens during the first months of the pandemic, which showed that 16.5%, 8.1% and 28.8% recognized moderate to severe depressive, stress and anxiety symptoms, respectively, in addition to the high percentage of concern about infecting family members (75%). Similarly, another study conducted between 30 January and 3 February 2020 in China with a total of 2091 participants found a prevalence of acute post-traumatic stress symptoms in 4.6% of the sample, associated with gender, date of report and location [29].

This trend pointing to the strong psychological impact of COVID is replicated cross-nationally. An example of this is the study by Asmundson and Taylor [30] in Canada, which showed a high level of public concern about becoming infected, despite the low number of cases found in that region. A study of 1143 families in Spain and Italy with children aged between 3 and 18 years found that 85.7% of parents had perceived changes in their children’s behavior and emotional state during the period of confinement, with boredom and concentration problems standing out [31]. In the case of the Spanish population, women, young people and those who lost their jobs showed the most symptoms, and among a sample of 3055, 25% showed mild to severe levels of anxiety, 41% depression and 41% stress [32]. On the other hand, there are studies that show that students have lower levels of anxiety than adults who are not students, as in the research carried out in the Romanian population with a sample of 759 people aged between 18 and 70 years [33].

In the case of the university population, the group on which this study focuses, previous research has shown that due to this being their vital moment of transition between academic and professional life, it is likely that they will experience high levels of stress, anxiety and depression [34]. The situation of confinement, in addition to all the above mentioned, caused them to face a more demanding training situation, both in terms of management, organization and the achievement of academic goals [35]. The major changes that threaten the socio-emotional and mental balance of this population in their transition to university [14], as well as the measures adopted by the educational institutions, have led to the maintenance of adequate levels of mental health. Furthermore, research carried out with this population in the initial stages after the state of alarm decreed in different universities shows levels that need to be monitored [36,37]. In this regard, the study by Odriozola-González et al. [38] of 2530 university students during the first weeks of confinement found that the levels of anxiety, depression and stress measured using the DASS-21 scale show that they have suffered a significant psychological impact, with the percentages obtained being 21.34% for anxiety, 34.19% for depression and 28.14% for stress, which show moderate to severe levels. Another study of 932 Spanish university students shows that this population experienced psychological problems, with higher rates among women and undergraduates than among men and graduate students; it also analyzes the most effective coping strategies, which were found to be reframing skills and daily routines [39].

On the other hand, previous studies before the adverse health situation, which relate EI to coping strategies and stress, show that in nursing students EI is positively related to problem-focused coping strategies and negatively related to stress [40]; the same is shown by Enns et al. [41], relating perceived stress to lower levels of EI; high levels of EI to a habitual use of adaptive coping strategies; and similarly, showing that these, together with maladaptive coping strategies, mediate between EI and stress in nursing students. In the case of a sample of 18–25-year-olds, the study conducted by Puigbó et al. [42] found the same results, with EI favoring emotional well-being, as it favors adaptive coping when faced with stress.

Given the significant impact on the psychological health of this population and the impact that this situation is expected to continue to have on university students [10,39,43], the purpose of this research is to analyze how EI, coping strategies and levels of anxiety, depression and stress have been affected by the situation generated by the pandemic. At the same time, we also analyzed the relationship between the variables considered, the personal circumstances that they have had during the confinement and the tendency to follow the measures and recommendations promoted by the Ministry of Health.

## 2. Materials and Methods

### 2.1. Participants

The sample participating in the study consisted of 567 students from the University of Jaén who were enrolled in education degrees. Its average age was M 21.08 (SD 4.13) and its sex distribution was 81% females and 19% males. This proportion is consistent with the sex distribution in education degrees in Spain [44]. Of the sample, 54% are in their first year, 28% in their second year, 9% in their third year and 9% in their fourth year. The distribution of the sample by degree corresponds to 27% of the social education degree, 23% of the infant education degree, 18% of the psychology degree and 16% of the primary education degree, with the remaining percentage belonging to degrees that do not reach 5% of the sample. 

### 2.2. Instruments

For the assessment of the different variables, three validated subscales and a fourth part which consists of a series of categorical questions were used.

The Wong and Law emotional intelligence scale (WLEIS) [45] was used to collect data on EI, which allows for the assessment of four dimensions (aelf and other emotion appraisal (SEA), use of emotion (UOE) and regulation of emotion (ROE)) through 16 items. Specifically, the Spanish version of Extremera et al. [46] was used, validated with a reliability of α = 0.91.

The Spanish version of the coping strategies inventory (CSI) [47] was used to obtain information on the coping strategies employed by the students, in its Spanish version with 40 items in a Likert-type scale format with an internal consistency of between 0.63 and 0.89. This instrument assesses eight primary coping strategies: (1) problem solving (PSR); (2) self-criticism (SCA); (3) emotional expression (EEM); (4) desiderative thinking (PDS); (5) social support (SSA); (6) cognitive restructuring (CR); (7) problem avoidance (PAD); (8) social withdrawal (SR).

The last scale used in the study is the depression anxiety stress scales (DASS-21) [48], which provides information on the psychological impact, anxiety, depression and stress of students, both their presence and intensity through three scales, one for each affective state; these are made up of seven Likert-type response items. The Spanish version has an internal consistency of 0.84 for depression, 0.70 for anxiety and 0.82 for stress.

Regarding the different subscales, categorical questions were presented which would allow insight into behaviors or situations carried out by the students during the pandemic period. The questions were as follows: Q1: Have you ever experienced a traumatic situation before the one generated by the COVID-19 virus? Q2: During the confinement caused as a consequence of the COVID-19 state of emergency, how are you experiencing the confinement? Q3: How many people are experiencing the confinement in your home; Q4: How often do you go out to the street? Q5: What is the purpose of these outings? Indicate as many options as appropriate. Q6: During these outings, what protective equipment do you use? Q7: How many times do you wash your hands per day? All possible response options were already fixed, so there was no possibility of an open-ended response. The response options for each question were: Q1: (a) yes, (b) no; Q2: (a) alone, (b) with family, (c) non-family company; Q3: from (a) 1 to, (b) 2, (c) 3, (d) 4, (e) 5, (f) 6, (g) 7, h)8; Q4: (a) less than twice a week, (b) more than twice a week, (c) once a day, (d) more than once a day; Q5: (a) shopping, (b) taking the dog out, (c) taking out the rubbish, (d) working, (e) assisting dependent people; Q6: (a) none, (b) gloves, (c) mask, (d) gloves and mask; Q7: (a) none, (b) one, (c) two to five times, (d) more than five times. Except for Q5, students could only choose one answer option for each of the questions.

### 2.3. Procedure

After the development of the final questionnaire with the different scales and questions, it was distributed from 5 April 2020 among the university students using the Google Form tool, which was available throughout the semester. For the administration of the link, the chats of the online teaching sessions and the institutional platform were used. The study was approved by the ethics committee of the University of Jaén (Reference: OCT.20/1.TES), following the Declaration of Helsinki [49]; therefore, participation in the study was anonymous and voluntary, with an informed consent form at the beginning of the study, and the conditions of the study had to be accepted in order to be able to access the questionnaire. 

### 2.4. Data Analysis

All analyses carried out in this work were performed with R and Jamovi software [50]. Prior to the analysis of the levels of the latent variables obtained through the self-report scales, we analyzed the psychometric properties of these scales in this population. For this purpose, a Confirmatory Factor Analysis (CFA) was performed using the popular R package lavaan [51]. We use Cronbach’s alpha and McDonald ω to analyze the internal consistency of the scales [52]. In order to make the results of the data obtained through the scales more sensitive, the raw data obtained through the scales were scaled by the factor loadings obtained in the CFAs [53]. Finally, a multiple regression analysis was performed with the predictor variables Q1–Q7 and the dependent variables measured through the different scales (EI, COP and DASS). The regressions were estimated using a general linear model fit by OLS (Ordinary Least Squares) through the *GMLj* package of Jamovi, where the categorical variables were considered dummy variables.

## 3. Results

### 3.1. Psychometric Analysis of the Scales Used

The data screening of the results obtained through the different scales used showed that our data did not breach the assumptions for the factorial treatment; however, this data analysis allowed us to know that our data did not have a multivariate normal distribution (Mardia’s Test *p* < 0.01).

A CFA was performed on each of the data from each of the three scales used. The results of this psychometric analysis for each of the scales are presented below. 

The results for the WLEIS-S scale showed an excellent fit [54], χ^2^ (98) = 528,481, *p* < 0.001, con CFI = 0.946, TLI = 0.934, SRMR = 0.107, RMSEA = 0.088 (RMSEA 90% CI (0.081, 0.096)) and very good reliability rates α = 0.896 and ω = 0.897. The CFA for CSI scale showed an excellent fit [55], χ^2^ (704) = 1,415,619, *p* < 0.001, con CFI = 0.953, TLI = 0.948, SRMR = 0.058, RMSEA = 0.042 (RMSEA 90% CI (0.039, 0.045)) and good reliability rates α = 0.795 and ω = 0.818. Finally, the CFA results for DASS-21 also showed an excellent fit [49], χ^2^ (186) = 205,669, *p* = 0.154, con CFI = 0.999, TLI = 0.999, SRMR = 0.038, RMSEA = 0.014 (RMSEA 90% CI (0.000, 0.024)) and excellent reliability rates α = 0.938 and ω = 0.938.

Table 1 presents the correlation matrix between the dependent variables after scaling. As can be seen, there is a positive and significant correlation between EI and COP and between COP and DASS, and a significant negative correlation between EI and DASS.

### 3.2. Multiple Regression Analysis

Finally, we performed a multiple regression analysis with the dependent variables obtained from the scales and with the predictor variables, the categorical questions included in the questionnaire (Sex, and Q1–Q7).

Table 2 shows the results of the multiple regression analysis represented by the effect of the ANOVA omnibus test for each dependent variable. Numerous significant effects emerged from this analysis.

Appendix A presents detailed descriptive results for the three variables according to the different response levels for each question. As we can see from the results shown in Table 2, the variable sex showed an effect for EI (with higher EI in males) and for DASS (with higher DASS in females). In the case of Q1, those staff who indicated that they had been through a traumatic situation showed higher COP and higher DASS. For question Q2, those students who went through the confinement with family showed higher levels of DASS, while those who went through it alone showed higher levels of COP, and those who went through it in non-family company showed lower levels of COP. Q4 only showed a COP effect with those who reported going out only once a day and they had the lowest COP levels. In question Q5, there was no effect on the factors associated with the number of purposes chosen as a response. On the other hand, we found effects associated with the types of purposes or behaviors for the EI variable, and for the DASS variable. Specifically, those students who indicated that their outings were for the purpose of buying or throwing away rubbish showed higher levels of EI. In the case of DASS, those students whose purposes were related to leisure showed lower levels of DASS. Q6 showed an effect on all three variables, with students who reported not using any protective measures showing lower levels of EI and higher levels of DASS, and those who reported using masks and gloves showing higher POPs. Finally, question Q7 only showed an effect on EI where those who reported not washing their hands at all times showed the highest EI score, while the lowest was shown by those who reported washing their hands once. 

## 4. Discussion

This study sought to analyze the relationship between EI, coping strategies and levels of depression, anxiety and stress in relation to the behaviors and situations developed by university students in the pandemic period. 

In line with previous research [14,56], the results obtained have found a positive relationship between EI and coping strategies and a negative relationship between EI and levels of anxiety, depression and stress. In this regard, EI may be associated with adaptive coping strategies in response to the burden of negative emotions that may have been produced by the pandemic and the confinement derived from it. The relationship between EI and coping has already been identified in the literature [40,41,42,57], and a significant relationship has also been found between coping strategies and levels of anxiety, depression and stress, unlike the study by Lopes and Nihei [58] conducted with Brazilian university students. Based on the results found, EI can be considered as a protective factor that is fundamental for dealing with various everyday or particular situations, such as that produced by COVID-19 which involves high levels of overload, and it is therefore of vital importance for teachers to take it into account. 

Accordingly, promoting the development and acquisition of soft skills such as EI or resilience should be a priority within curricula, with the intention of combating episodes of academic stress or low self-esteem, given their close relationship with academic performance, not only in the university stage [59], but also in previous educational stages, due, among other issues, to the importance of accumulated experience in the acquisition of these skills [40,60].

In the analysis of the difference between men and women, the results obtained are consistent with other studies carried out around the world during this period, where the highest levels of anxiety, depression and stress are found in women, with the effect that it is this population, together with young adults, who have been most affected by depressive symptoms derived from COVID-19 [4,61,62].

Relating the variables studied through the scales with the categorical questions about the situations and behaviors performed by the students during the COVID-19 quarantine, we found that the students who had already experienced a previous traumatic situation were those who presented a greater repertoire of coping strategies, as well as higher levels of anxiety, depression and stress, in line with previous studies carried out in China, Europe and the United States [30,63,64]. In relation to the company during the quarantine, the results show that those students who were with their family had higher levels of stress, depression and anxiety; on the other hand, those who spent the quarantine alone showed more coping strategies compared to those who were accompanied by non-family members who showed poorer coping strategies. It is because of these data that the family can be foreseen as a source of stress in situations of imprisonment, among other aspects derived from the situations that the family units had to face (economic problems, reconciliation of children’s education and teleworking, additional personal burdens, etc.), which can affect the health and mental wellbeing of all family members in general and each one of them in particular [65,66]. In terms of the reason for going out, students who indicated more leisure-related purposes showed lower levels of anxiety, depression and stress, with students who ticked the options “shopping” or “taking out the rubbish” showing higher levels of EI compared to the other options (taking the dog out, working or helping dependents). This could be due to the fact that students who do more leisure activities show less anxiety, depression and stress. When analyzing the different types of protective equipment used during the outings, it was found that those who did not use any type of protection had a lower level of EI and higher levels of depression, anxiety and stress; and those who used both masks and gloves also had lower levels of these three parameters and higher levels of coping strategies. The conclusion is that students who are less aware of the consequences of COVID-19 show lower levels of EI, and therefore higher levels of DASS. Finally, the data obtained regarding hand washing reveal unexpected results, since it was assumed that the subjects with higher EI would take into account the rules of hygiene to combat the virus, as opposed to the result obtained, which showed higher EI in those students who never wash their hands. However, on examining these results in more detail, it can be seen that they are due to the low number of subjects (7) and the low variability, which leads to the conclusion that it is those students who wash their hands more than five times a day who show higher levels of EI.

From the results shown, it appears that subjects with high EI are those who use more protective measures and display coping strategies to deal with problems arising from the pandemic. These results are in line with previous studies by Enns et al. [41], Matthews et al. [67] and Vintila et al. [33], who reported that emotionally intelligent subjects were able to implement more effective coping strategies in response to stressful and/or life-threatening events, either through direct management of the stressor (e.g., wearing gloves and masks), or through seeking opportunities to develop and learn in such adverse circumstances (e.g., taking advantage of partner/family time, etc.).

## 5. Conclusions

In reference to the practical implications of this study, we found that there are numerous studies that advocate the inclusion of EI in both educational experiences and programs [68,69]; therefore, the results obtained support the development of prevention and intervention programs related to EI, which would improve the use of coping strategies in the COVID-19 period and in subsequent periods of a similar nature, in order to reduce the levels of anxiety, depression and stress.

Finally, this study is not free of limitations that refer, firstly, to the design, due to its cross-sectional nature, which makes it impossible to establish causality between the variables studied; secondly, in reference to the sample, which does not allow us to generalize the results, given the specific nature of the grades for which results have been obtained and the low number of male participants; and finally, the lack of studies carried out that analyze the moderating effect between EI, COP and DASS during the pandemic situation in the university student population, which prevents the results obtained from being compared with other similar studies. Therefore, further research should consider a longitudinal design that would provide a deeper understanding of the associations between the variables analyzed, as well as collecting data from a wider range of degree programs in order to generalize the results for the university student population in general.

## Figures and Tables

**Table 1:** Correlation Matrix.

	Mean (SD)	EI	COP
EI	*2.63 (0.68)*		
COP	*1.99 (0.261)*	0.211 ***	
DASS	0.99 (0.51)	−0.264 ***	0.215 ***

EI = Emotional intelligent, COP = Coping, DASS = Depression, anxiety and stress *** *p* < 0.001.

**Table 2:** Multiple regression analysis for each dependent variable.

Dependent Variables		Sex	Q1	Q2	Q3	Q4	Q5	Q6	Q7
EI	SS	1743	0.799	0.369	2.234	2.373	22.591	4.918	6.791
	*df*	1	1	2	9	3	44	3	3
	*F*	5.437	2.466	0.567	0.762	2.455	1.663	5.160	7.202
	*p*	0.020	0.117	0.567	0.652	0.062	0.006	0.002	<0.001
	*η²_p_*	0.010	0.004	0.002	0.012	0.013	0.124	0.027	0.037
COP	SS	0.009	0.560	0.428	0.373	0.623	2.874	0.623	0.122
	*df*	1	1	2	9	3	44	3	3
	*F*	0.133	8.347	3.174	0.606	3.091	0.958	3.093	0.596
	*p*	0.716	0.004	0.043	0.792	0.027	0.551	0.027	0.618
	*η²_p_*	0.000	0.015	0.011	0.010	0.016	0.076	0.016	0.003
DASS	SS	2.651	5.461	2.218	3.373	1.746	12.831	4.443	1.156
	*df*	1	1	2	9	3	44	3	3
	*F*	10.422	21.703	4.299	1.447	2.245	1.129	5.822	1.480
	*p*	0.001	<0.001	0.014	0.165	0.082	0.269	<0.001	0.219
	*η²_p_*	0.018	0.037	0.015	0.023	0.012	0.088	0.030	0.008

EI = Emotional intelligent, COP = Coping, DASS = Depression, anxiety and stress. Q1: Have you ever experienced a traumatic situation before the one generated by the COVID-19 virus? Q2: During the confinement caused as a consequence of the COVID-19 state of emergency, how are you experiencing the confinement? Q3: How many people are experiencing the confinement in your home? Q4: How often do you go out to the street? Q5: What is the purpose of these outings? Indicate as many options as appropriate. Q6: During these outings, what protective equipment do you use? Q7: How many times do you wash your hands per day?

## Data Availability

Data are available on justified request to the corresponding author.

## Appendix A

**Table A1:** Descriptive results for the three variables according to the different response levels for each question.

Factors	Levels		DASS	COP	EI
Sex	Man		n = 106, M = 0.85 (0.47)	n = 106, M = 1.99 (0.24)	n = 106, M = 2.75 (0.47)
	Woman		n = 461, M = 1.03 (0.51)	n = 461, M = 2.00 (0.27)	n = 461, M = 2.61 (0.59)
Q1	Yes		n = 157, M = 1.15 (0.53)	n = 157, M = 2.04 (0.24)	n = 157, M = 2.57 (0.56)
	No		n = 403, M = 0.93 (0.49)	n = 403, M = 1.97 (0.27)	n = 403, M = 2.65 (0.57)
Q2	Alone		n = 8, M = 0.81 (0.37)	n = 8, M = 2.04 (0.15)	n = 8, M = 2.60 (0.56)
	With the family		n = 498, M = 1.01 (0.51)	n = 498, M = 2.00 (0.26)	n = 498, M = 2.62 (0.58)
	Non-family company		n = 54, M = 0.81 (0.53)	n = 54, M = 1.91 (0.26)	n = 54, M = 2.71 (0.52)
Q3	One		n = 10, M = 0.88 (0.34)	n = 10, M = 2.06 (0.16)	n = 10, M = 2.64 (0.64)
	Two		n = 71, M = 0.84 (0.50)	n = 71, M = 1.98 (0.25)	n = 71, M = 2.64 (0.48)
	Three		n = 134, M = 1.04 (0.45)	n = 134, M = 2.00 (0.26)	n = 134, M = 2.58 (0.56)
	Four		n = 221, M = 1.00 (0.55)	n = 221, M = 2.00 (0.25)	n = 221, M = 2.61 (0.58)
	Five		n = 100, M = 1.03 (0.48)	n = 100, M = 1.98 (0.30)	n = 100, M = 2.74 (0.60)
	Six		n = 15, M = 1.07 (0.54)	n = 15, M = 1.92 (0.30)	n = 15, M = 2.49 (0.61)
	Seven		n = 6, M = 0.90 (0.89)	n = 6, M = 2.07 (0.07)	n = 6, M = 2.84 (0.93)
	Eight		n = 2, M = 0.43 (0.61)	n = 2, M = 1.74 (0.27)	n = 2, M = 2.80 (0.60)
Q4	Less 2 times week		n = 230, M = 1.04 (0.53)	n = 230, M = 2.02 (0.26)	n = 230, M = 2.64 (0.58)
	Once a day		n = 126, M = 0.98 (0.52)	n = 126, M = 1.94 (0.27)	n = 126, M = 2.55 (0.56)
	More than once a day		n = 72, M = 1.02 (0.50)	n = 72, M = 2.02 (0.27)	n = 72, M = 2.58 (0.57)
	More 2 times week		n = 132, M = 0.90 (0.47)	n = 132, M = 1.98 (0.24)	n = 132, M = 2.73 (0.56)
Q5	Buy	Yes	n = 426, M = 0.99 (0.51)	n = 426, M = 1.99 (0.27)	n = 426, M = 2.67 (0.58)
		No	n = 134, M = 1.00 (0.53)	n = 134, M = 1.99 (0.22)	n = 134, M = 2.50 (0.53)
	Take out dog	Yes	n = 359, M = 0.98 (0.54)	n = 359, M = 1.99 (0.26)	n = 359, M = 2.64 (0.56)
		No	n = 201, M = 1.01 (0.45)	n = 201, M = 2.00 (0.27)	n = 201, M = 2.62 (0.59)
	Littering	Yes	n = 251, M = 1.02 (0.50)	n = 251, M = 1.99 (0.26)	n = 251, M = 2.58 (0.56)
		No	n = 309, M = 0.96 (0.52)	n = 309, M = 2.00 (0.27)	n = 309, M = 2.67 (0.57)
	Leisure	Yes	n = 537, M = 1.00 (0.51)	n = 537, M = 1.99 (0.26)	n = 537, M = 2.63 (0.57)
		No	n = 23, M = 0.82 (0.61)	n = 23, M = 2.04 (0.19)	n = 23, M = 2.55(0.55)
	Work	Yes	n = 468, M = 1.00 (0.51)	n = 468, M = 1.99 (0.27)	n = 468, M = 2.64 (0.57)
		No	n = 92, M = 0.96 (0.54)	n = 92, M = 2.00 (0.24)	n = 92, M = 2.58 (0.59)
	Dependent assistance	Yes	n = 74, M = 0.95 (0.51)	n = 74, M = 2.00 (0.23)	n = 74, M = 2.65 (0.56)
		No	n = 486, M = 1.00 (0.51)	n = 486, M = 1.99 (0.27)	n = 486, M = 2.63 (0.57)
Q6	None		n = 31, M = 1.17 (0.39)	n = 31, M = 1.99 (0.28)	n = 31, M = 2.32 (0.42)
	Gloves		n = 20, M = 0.92 (0.51)	n = 20, M = 1.98 (0.25)	n = 20, M = 2.64 (0.44)
	Face mask		n = 316, M = 0.92 (0.51)	n = 316, M = 1.97 (0.27)	n = 316, M = 2.60 (0.56)
	Gloves and mask		n = 193, M = 1.08 (0.51)	n = 193, M = 2.04 (0.24)	n = 193, M = 2.72 (0.60)
Q7	None		n = 7, M = 0.92 (0.61)	n = 7, M = 1.98 (0.20)	n = 7, M = 2.82 (0.38)
	One		n = 25, M = 1.10 (0.44)	n = 25, M = 1.95 (0.29)	n = 25, M = 2.35 (0.57)
	2 to 5 times		n = 294, M = 0.95 (0.51)	n = 294, M = 1.98 (0.25)	n = 294, M = 2.56 (0.56)
	More than 5 times		n = 234, M = 1.03 (0.52)	n = 234, M = 2.01 (0.27)	n = 234, M = 2.75 (0.56)

For each dependent variable and each level of the questions analyzed, the number of cases (n), the mean (M) and the standard deviation of the mean are presented first in brackets. DASS = Depression, anxiety and stress, COP = coping, EI = emotional intelligent. Q1: Have you ever experienced a traumatic situation before the one generated by the COVID-19 virus? Q2: During the confinement caused as a consequence of the COVID-19 State of Emergency, how are you experiencing the confinement? Q3: How many people are experiencing the confinement in your home? Q4: How often do you go out to the street? Q5: What is the purpose of these outings? Indicate as many options as appropriate. Q6: During these outings, what protective equipment do you use? Q7: How many times do you wash your hands per day?
